# LncRNA NORAD facilitates oral squamous cell carcinoma progression by sponging miR-577 to enhance TPM4

**DOI:** 10.1186/s13062-021-00299-2

**Published:** 2022-01-06

**Authors:** Change Qi, Jianwei Liu, Pengnv Guo, Yali Xu, Jing Hu, Xiaomei Han

**Affiliations:** 1 Department of Oral Implantology, Affiliated Hospital of Chifeng University, No. 4, Section 3, East Yuanlin Road, Chifeng, 024000 Inner Mongolia China; 2 Department of Oral and Maxillofacial Surgery, Affiliated Hospital of Chifeng University, Chifeng, 024000 Inner Mongolia China

**Keywords:** NORAD, miR-577, TPM4, Oral squamous cell carcinoma

## Abstract

**Background:**

Long non-coding RNAs (lncRNAs) have been reported to be vital factors to affect the expression of genes and proteins. Also, it has been proved that the abnormal expression or mutation of lncRNAs stands as a signal of metastasis and proliferation of cancer. Nevertheless, the majority of lncRNAs still need to be explored in abundant cancers especially in oral squamous cell carcinoma (OSCC).

**Methods:**

RT-qPCR assays were applied to test the expression of RNAs. Mechanism assays were performed to verify the combination among NORAD, TPM4 and miR-577. Also, functional assays were conducted to verify the function of RNAs on OSCC cells.

**Results:**

LncRNA NORAD was highly expressed in OSCC tissues and cells. NORAD silencing repressed the biological behaviors of OSCC cells. MiR-577 was found in OSCC with low expression, and RIP assays illustrated that NORAD, miR-577 and TPM4 coexisted in RNA-induced silencing complexes. Rescue assays proved that the overexpression of TPM4 could recover the effect of NORAD silencing on OSCC progression.

**Conclusions:**

It was revealed that NORAD functioned as a tumor promoter to sponge miR-577 thus elevating TPM4 in OSCC, which indicated that NORAD was worthy to be studied as a target for the treatment of OSCC.

**Supplementary Information:**

The online version contains supplementary material available at 10.1186/s13062-021-00299-2.

## Background

Oral squamous cell carcinoma (OSCC) is a typical cancer belonging to head and neck cancer with growing incidence [1]. Although great progress has been made in its treatment, the recurrence rate remains extremely high on account of metastasis [2]. Thus, it is necessary to have a deep understanding of the molecular mechanism in OSCC development in order to improve the therapy result as well as to come up with more efficient treatment courses. Recently, competing endogenous RNA (ceRNA) regulatory system has been reported in substantial articles where lncRNAs plays as a sponge of microRNAs (miRNAs) to regulate their downstream target in cancer [3–5]. PTENP1 has been reported as a ceRNA in breast cancer by sponging miR-19b to regulate PTEN [6]. HMGA1 pseudogene 7 has been discovered to induce miR-483 and miR-675 enhancement via activating Egr1 in ceRNA mechanism [7]. NORAD is the abbreviation of Noncoding RNA Activated by DNA Damage and has been proved to promote the progression of colorectal cancer [8]. However, the role of NORAD in OSCC has not been clarified and how NORAD may exert its function in ceRNA network still needs to be explained.

Mounting evidence has suggested that miRNAs are significant regulators in gene expression which can contribute to tumor development or restrict tumor progression [9–12]. MiR-133a-3p has been demonstrated as a tumor inhibitor in OSCC via repressing COL1A1 [13]. MiR-1271 has been introduced to inhibit the metastasis and proliferation of OSCC by targeting ALK [14]. MiR-577 has been found to exert inhibitory function in papillary thyroid carcinoma [15] and glioblastoma tumor [16]. Nonetheless, the function of miR-577 in OSCC remains unknown to us.

In a word, the purpose of our study was to analyze the role of NORAD in OSCC and further explore the ceRNA regulatory mechanism of NORAD, hoping to provide novel therapeutic options for the future NORAD treatment.

## Results

### NORAD was highly expressed in OSCC cells and boosted OSCC progression

**Fig. 1:**
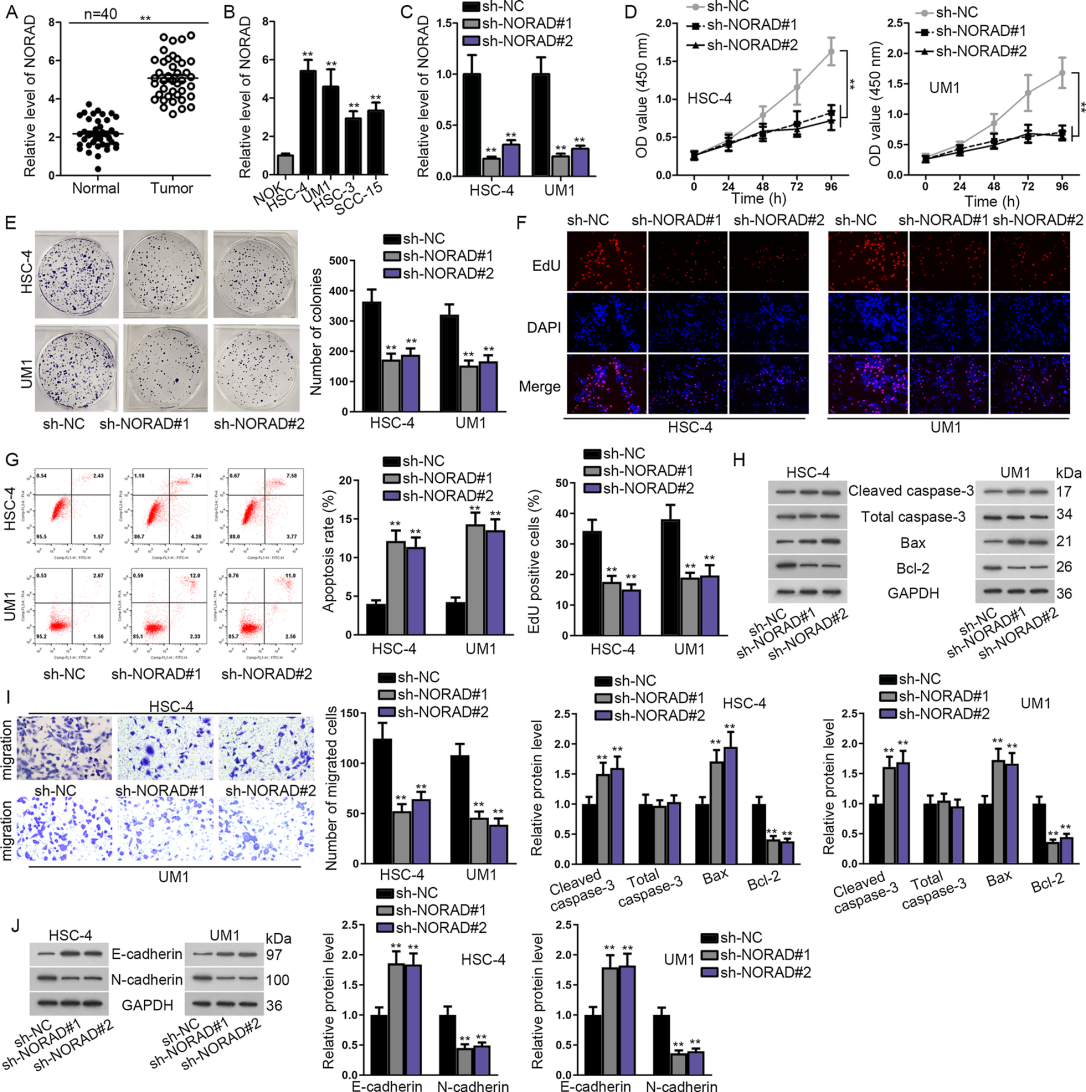
NORAD was highly expressed in OSCC cells and boosted OSCC progression. **a** NORAD expression was examined in OSCC tissue and adjacent normal tissue by RT-qPCR assay. **b** NORAD expression was tested in OSCC cell lines (HSC-4, UM1, HSC-3 and SCC-15) and normal keratinocyte (NOK) by RT-qPCR assay. **c** RT-qPCR assay was conducted to detect the expression of NORAD in HSC-4 and UM1 cell transfected with sh-NORAD#1/#2. **d**–**f** The ability of cell proliferation upon NORAD silencing was detected by CCK-8, colony formation and EdU assays. **g** The apoptosis rate of cells upon NORAD silencing was assessed by flow cytometry analysis. **h** The relevant protein level of apoptosis in cells transfected with sh-NORAD#1/#2 was analyzed by western blot. **i** Cell migration upon NORAD silencing was estimated by Transwell assays. **j** Western blot assay was applied to measure the protein level of EMT-associated proteins in cells transfected with sh-NORAD#1/#2. **P < 0.01

To investigate the role of NORAD in OSCC, RT-qPCR assays were firstly used to detect the expression of NORAD in tissues. The results indicated that NORAD was highly expressed in OSCC tissues compared with that in normal adjacent tissue (Fig. 1a) and the data from online database was presented (Additional file 1: Fig. S1B). We also measured the expression of NORAD in OSCC cell lines (HSC-4, UM1, HSC-3 and SCC-15) and normal keratinocyte cell line (NOK). The results showed that the expression of NORAD was high in OSCC cell lines in comparison with that in NOK (Fig. 1b). Among these cells, higher expression of NORAD was displayed in HSC-4 and UM1 cells than the others, so HSC-4 and UM1 were chosen for the following experiments. To detect the effect of sh-NORAD#1 and sh-NORAD#2 which were transfected into cells, the expression of NORAD was examined by RT-qPCR assays. According to the results, it was manifested that NORAD expression was reduced after the transfection of sh-NORAD#1 and sh-NORAD#2 compared with that with normal control (Fig. 1c). After that, the capacity of cell proliferation was tested by CCK-8, colony formation and EdU assays and the results suggested that cell proliferation ability was dramatically declined by NORAD knockdown in comparison with normal control group (Fig. 1d–f). What’s more, the rate of cell apoptosis was prominently lifted by the knockdown of NORAD compared with that of normal control group (Fig. 1g). The associated protein of apoptosis was estimated by western blot assay and the results showed that the expression of cleaved caspase-3 and Bax were remarkably elevated by the knockdown of NORAD while the expression of Bcl-2 was obviously decreased (Fig. 1h). The ability of migration was conspicuously dropped by the knockdown of NORAD in contrast with that of normal control group (Fig. 1i). After that, the expression of relevant proteins of epithelial mesenchymal transition (EMT) was detected by western blot assay and the result presented that E-cadherin expression was increased while that of N-cadherin was diminished (Fig. 1j). Taken together, NORAD was found to be highly expressed in OSCC tissues and cells and it hastened the progression of OSCC cells.

### NORAD bound to miR-577 in OSCC

**Fig. 2:**
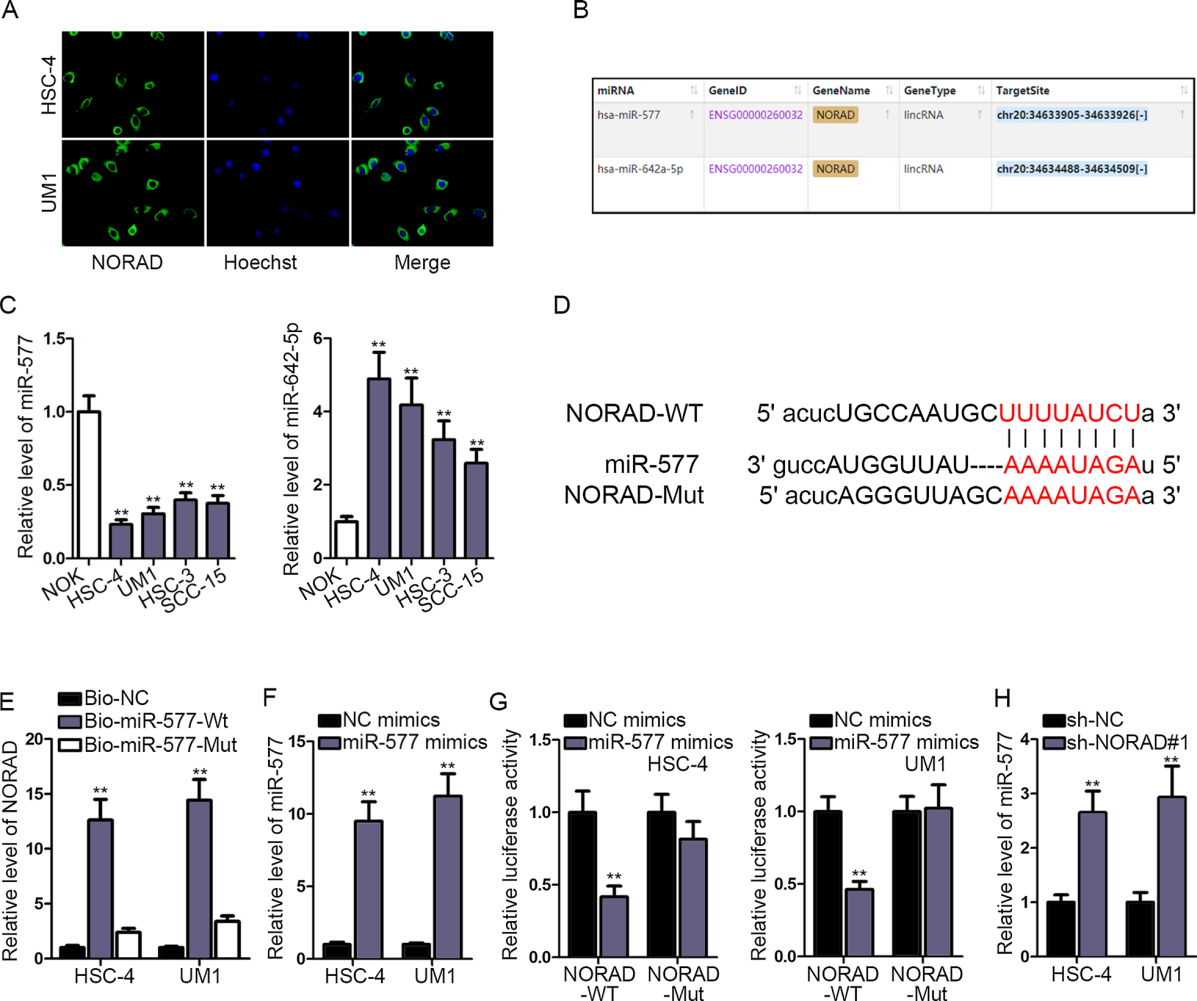
NORAD bound to miR-577 in OSCC. **a** FISH assays were conducted to evaluate the distribution of NORAD in nucleus and cytoplasm. **b** Possible miRNAs which could bind to NORAD were predicted by starBase database. **c** RT-qPCR assays were used to examine the expression of miR-577 and miR-642-5p in OSCC cell lines **d** Bioinformatics analysis was adopted to predict the potential binding sites between miR-577 and NORAD. **e** RNA pull down assay was performed to verify whether miR-577 could bind to NORAD. **f** RT-qPCR assay was performed to detect the overexpression efficiency of miR-577 mimics. **g** Luciferase reporter assays were carried out to verify whether miR-577 could bind to NORAD. **h** The expression of miR-577 in HSC-4 and UM1 cells transfected with sh-NORAD#1 was estimated by RT-qPCR assay. **P < 0.01

To investigate how NORAD functioned as a ceRNA in OSCC, we adopted FISH assay to ascertain the subcellular localization of NORAD and the result showed that NORAD was mainly distributed in the cytoplasm (Fig. 2a). After that, starBase (http://starbase.sysu.edu.cn) was used to find out the potential miRNAs which had binding sites with NORAD and the results unveiled that miR-577 and miR-642a-5p were the potential ones (Fig. 2b). Then, RT-qPCR assays were carried out to test the expression of miR-577 and miR-642a-5p in OSCC cell lines and the result exhibited that miR-577 was found in OSCC cells with low expression while miR-642a-5p expression was highly expressed (Fig. 2c). Hence, miR-577 was selected. Also, the expression of miR-577 in tumor and normal adjacent tissues was detected by RT-qPCR assay and searched online (Additional file 1: Fig. S1 A, B). After that, bioinformatics analysis was applied and the putative binding sites between miR-577 and NORAD were predicted (Fig. 2d). Then, RNA pull down assays were conducted and we observed that biotinylated miR-577-WT could pull down NORAD while no distinct change could be seen in biotinylated miR-577-Mut (Fig. 2e). After that, miR-577 mimics was transfected into HSC-4 and UM1 cells and RT-qPCR assay were performed to measure the expression of miR-577. As shown by the result, the expression of miR-577 in cells was increased after the transfection of miR-577 mimics (Fig. 2f). Next, luciferase reporter assays were carried out and the results displayed that the activity of plasmid built with NORAD-WT was obviously declined by miR-577 mimics compared with normal control group while no evident change could be found in plasmids set with NORAD-Mut (Fig. 2g). Therefore, NORAD could bind to miR-577. Then, HSC-4 and UM1 cells were transfected with sh-NORAD#1 and the expression of miR-577 was estimated by RT-qPCR assay. The results delineated that miR-577 expression was elevated by the knockdown of NORAD (Fig. 2h). To summarize, NORAD could bind to miR-577 and had a negative correlation with miR-577.

### TPM4 was the downstream target of miR-577

**Fig. 3:**
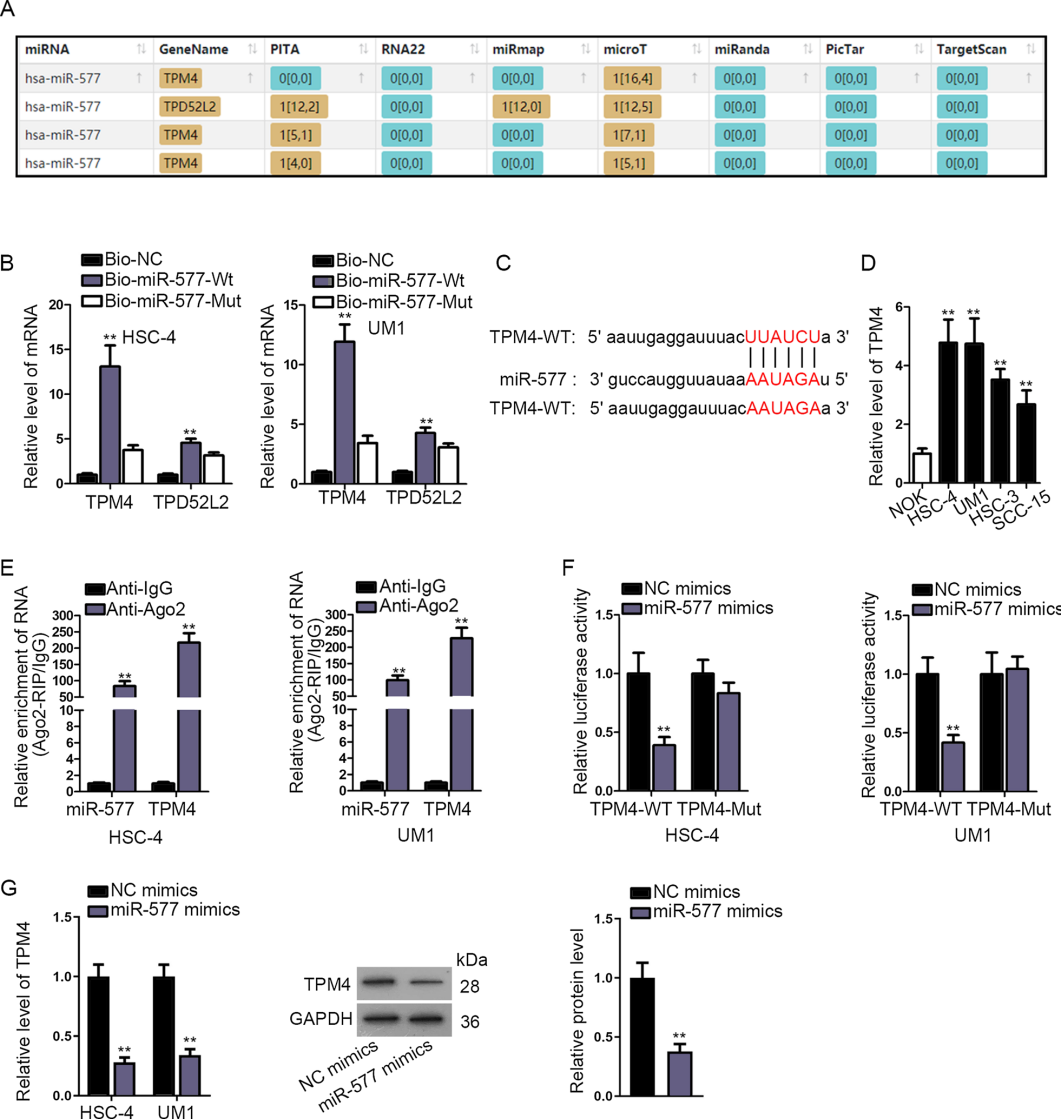
TPM4 was the downstream target of miR-577. **a** StarBase database was utilized to predict the potential mRNAs which could bind to miR-577. **b** RNA pull down assays were carried out to prove whether miR-577 could bind to TPM4. **c** Bioinformatics analysis was adopted to predict the binding sites between miR-577 and TPM4. **d** The expression of TPM4 was tested in OSCC cell lines. **e** RIP assay was used to confirm whether miR-577 and TPM4 coexisted in RISCs. **f** Luciferase reporter assays were performed to verify whether miR-577 could bind to TPM4. **g** The expression and protein level of TPM4 in cells transfected with miR-577 mimics was examined by RT-qPCR and western blot assays. **P < 0.01

To further unveil the role of NORAD in ceRNA network, we searched starBase database to find out the downstream target of miR-577. The result showed that TPM4 and TPD52L2 could be the potential ones (Fig. 3a). RNA pull down assays were then carried out and the results revealed that TPM4 could bind to miR-577 while no binding sign could be observed in the TPD52L2 group (Fig. 3b). Moreover, the binding sites between miR-577 and TPM4 were predicted by bioinformatics analysis (Fig. 3c). Then, we measured the expression of TPM4 in OSCC cell lines and the results depicted that TPM4 was highly expressed (Fig. 3d). Also, the expression of TPM4 in tumor and normal adjacent tissues was detected by RT-qPCR assay and searched online (Additional file 1: Fig. S1 A, B). Then, RIP assays were performed and we found both miR-577 and TPM4 were enriched in Ago2 while no change was seen in IgG, which meant that miR-577 and TPM4 coexisted in RISCs (Fig. 3e). Furthermore, luciferase reporter assay was conducted and the result showed that miR-577 could bind to TPM4 (Fig. 3f). After that, miR-577 mimics were transfected into cells and we used RT-qPCR and western blot assays to detect the expression and protein level of TPM4. The result revealed that TPM4 expression was declined by miR-577 mimics (Fig. 3g). In short, TPM4 was the downstream target of miR-577 and had a negative correlation with miR-577.

### NORAD accelerated OSCC progression by targeting miR-577/TPM4

**Fig. 4:**
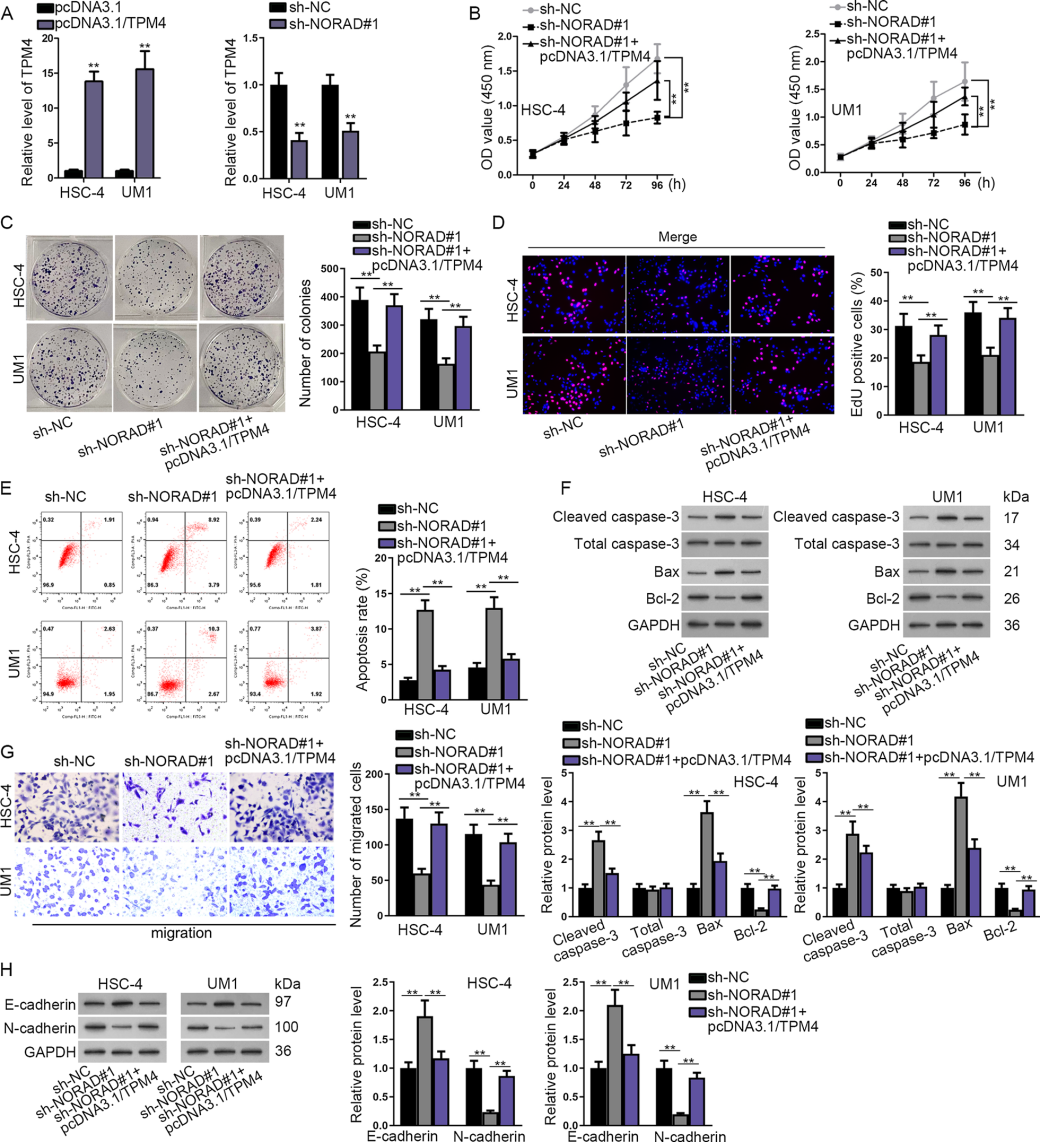
NORAD accelerated OSCC progression by targeting miR-577/TPM4. **a** The overexpression efficiency of pcDNA3.1/TPM4 in OSCC cells and TPM4 expression in cells transfected with sh-NORAD#1 were examined by RT-qPCR assays. **b**–**d** The proliferation of cells co-transfected with sh-NORAD#1 and pcDNA3.1/TPM4 was detected by CCK-8, colony formation and EdU assays. **e** The apoptosis rate of cells co-transfected with sh-NORAD#1 and pcDNA3.1/TPM4 was assessed by flow cytometry analysis. **f** Western blot assay was carried out to measure the protein level of apoptosis-associated proteins in cells co-transfected with sh-NORAD#1 and pcDNA3.1/TPM4. **g** Transwell assays were carried out to estimate the migration ability of cells co-transfected with sh-NORAD#1 and pcDNA3.1/TPM4. (H) Western blot assay was utilized to measure the protein level of EMT-associated proteins. **P < 0.01

To find out whether TPM4 took part in the process of OSCC development regulated by NORAD, rescue assays were carried out by us. At first, we transfected pcDNA3.1/TPM4 into HSC-4 and UM1 cells and used RT-qPCR assay to measure the expression of TPM4. The results presented that the expression of TPM4 was lifted by pcDNA3.1/TPM4 (Fig. 4a). Also, we detected the expression of TPM4 in OSCC cells transfected with sh-NORAD#1 and found a positive correlation between the expression of TPM4 and NORAD. According to the results shown by CCK-8, colony formation and EdU assays, the cell proliferation capacity was impaired by the knockdown of NORAD while the effect induced by sh-NORAD#1 was partially recovered by the co-transfection of pcDNA3.1/TPM4 (Fig. 4b–d). The apoptosis rate of cells which was increased by the knockdown of NORAD could also be partially reversed by the co-transfection of pcDNA3.1/TPM4 and the same result could be seen in the level of apoptosis-associated proteins (Fig. 4e, f). What’s more, the ability of migration decreased by NORAD silencing could be partially reversed by the co-transfection of TPM4 (Fig. 4g). The elevated expression of E-cadherin as well as the decreased expression of N-cadherin by the knockdown of NORAD could be partially recovered after the co-transfection of pcDNA3.1/TPM4 (Fig. 4h). To sum up, NORAD promoted the biological behaviors of OSCC cell by sponging miR-577 to enhance TPM4.

## Discussion

OSCC is a kind of cancer with high occurrence among the white [17] and it can bring a lot of pain to both the mind and body of the patients. A large quantity of researches has reported that lncRNAs are the crucial regulators of cancer growth [18–20]. LncRNA CC3 has been reported to enhance metastasis of cervical cancer via elevating Slug expression [21]. DILC has been found to modulate liver cancer stem cells through IL-6/STAT3 axis [22]. In our study, NORAD was highly expressed in OSCC tissues and cells, which was consistent with the finding that NORAD had high expression in cervical cancer [23]. Also, we found that the knockdown of NORAD suppressed the cell proliferation and migration of OSCC while enhancing the rate of apoptosis. Thus, we judged that NORAD was a tumor promoter in OSCC cells.

Recently, ceRNA network has become the limelight to study the cancer development and progression [24–27]. LncRNAs have been reported to bind to miRNAs via competing with mRNAs so that mRNAs could be released and participate in protein translation thus exerting their function in cancer. SNHG16 has been introduced to assist cell migration in breast cancer by sponging miR-98 to regulate E2F5 [28]. NEAT1 has been discovered to be a tumor promoter in ovarian cancer by sponging miR-194 to regulate ZEB1 expression [29]. PVT1 has been discovered as a ceRNA to promote progression of cervical cancer by sponging niR-424 [30]. In this study, miR-577 was found to have binding sites with NORAD and RNA pull down and luciferase reporter assays were used to illustrate that miR-577 could bind to NORAD. And miR-577 expression was negatively regulated by NORAD expression. TPM4 was confirmed to be the downstream target of miR-577 and it was highly expressed in OSCC cell lines. In rescue assays, we found that the overexpression of TPM4 could countervail the effect caused by NORAD knockdown. That means, NORAD acted as a ceRNA of miR-577 to enhance TPM4.

MiRNAs have been regarded as vital participators in the progression of multiple cancers [31, 32]. Even miR-195 has been treated as a therapeutic biomarker among breast cancer patients [33]. Numerous articles have indicated that miRNAs are the active factors to affect the growth of various cancers. For example, miR-126-3p has been found as a tumor inhibitor in thyroid cancer [34]. In our study, we found miR-577 in OSCC cell lines with low expression, which was also found in breast cancer [35]. And it negatively regulated the expression of TPM4. What’s more, miR-577 functioned as a bridge between NORAD and TPM4 to exert the effectiveness of NORAD/miR-577/TPM4 axis in regulating the biological behaviors of OSCC cells.

## Conclusions

In conclusion, the data from our studies revealed that NORAD was a tumor promoter and it accelerated the progression of OSCC by targeting miR-577/TPM4 axis. Knockdown of NORAD could restrict the biological behaviors in OSCC, which may provide a new sight for the therapy of OSCC. The shortcoming of our studies was the lack of in vivo experiments and clinical analysis. We will concentrate on those two aspects in the following studies in the coming future.

### Limitations

According to the OSCC tissue data from the TCGA database, there existed no significant difference in the expression of NORAD/miR-577 in cancer tissues and normal tissues. Therefore, more data from other public databases were required to support our research. In addition, the focusing point of the present study was to unveil the functions of NORAD on the progression of OSCC cells while the effect of NORAD on specific biological behaviors were not studied in detail and that served as a major limitation of our study.

## Materials and methods

### Tissue samples

Forty paired samples of OSCC tissues and adjacent healthy tissues were attained from patients with OSCC hospitalized at XXXX. Written informed consents had been acquired from patients and none of the patients underwent chemotherapy or radiotherapy prior to resection. After surgery, tissues were frozen in liquid nitrogen and stored at − 80 °C. This research protocol was licensed by the Ethics Committee of Affiliated Hospital of Chifeng University.

### Cell culture

Normal oral keratinocyte (NOK) and OSCC cells (HSC-4, UM1, HSC-3 and SCC-15) were acquired from the American Type Culture Collection (ATCC; Manassas, VA, USA). Cells were kept in DMEM (Gibco, Grand Island, NY, USA) adding 10% FBS (Gibco) plus antibiotics (Gibco) in a humidified atmosphere of 5% CO_2_ at 37 °C.

### Cell transfection

Specific shRNAs against NORAD (sh-NORAD#1 and sh-NORAD#2) and their corresponding NC (sh-NC), along with the pcDNA3.1 vector targeting TPM4 and the empty vector, were gained from Genechem (Shanghai, China). Simultaneously, miR-577 mimics and NC mimics were gained from GenePharma (Shanghai, China). HSC-4 or UM1 cells were selected for the transfection with each of these plasmids through Lipofectamine 3000 (Invitrogen, Carlsbad, CA, USA).

### Quantitative real-time RT-PCR (RT-qPCR)

With the application of Trizol (Invitrogen), total RNA was isolated and converted to cDNA by using the Revert Aid First Strand cDNA Synthesis Kit (Thermo Fisher Scientific, Waltham, MA, USA). The cDNA samples were assayed by qRT-PCR with the SYBR Premix Ex Taq Kit (Takara, Tokyo, China) and Stratagene Mx3000P (Agilent Technology, Austin, TX, USA). The expression levels of genes were normalized to the expression level of GAPDH or U6 as per 2^−ΔΔCt^ method.

### Cell counting kit-8 (CCK-8) assay

Cell viability was evaluated through Cell Counting Kit-8 (CCK-8) analysis according to the manufacturer’s guidelines. Transfected HSC-4 or UM1 cells were cultured in 96-well culture dishes. After that, 10 µl of CCK-8 solution (Solarbio, Beijing, China) was added to each well upon adherence, and further incubated at 37 °C with 5% CO_2_ for 2 h. Absorbance of samples was explored at 450 nm via the MRX II microplate reader (Dynex, Chantilly, VA, USA).

### Colony formation assay

As for colony formation assay, transfected HSC-4 or UM1 cells were placed into 6-well plates for 14 days of thermostatic culture. Upon this, colonies were gained and washed by using PBS (Sigma-Aldrich, St. Louis, MO, USA) before being immobilized for 15 min using paraformaldehyde (PFA; Sigma-Aldrich) and dyed for 10–30 min in crystal violet (Sigma-Aldrich) at room temperature. The optical microscope (Nikon, Tokyo, Japan) was applied for observing and counting the colonies.

### 5-Ethynyl-20-deoxyuridine (EdU) incorporation assay

The EdU assay kit (RiboBio, Guangdong, China) was applied for the exploration of cell proliferation. Transfected HSC-4 or UM1 cells were inoculated into 96-well plates overnight, followed by being incubated for 2 h with EdU solution. Cells were sequentially fixed for 30 min utilizing 4% PFA and incubated for 5 min with glycine (Sigma-Aldrich). For the next step, cells were permeabilized for 10 min using PBS of 0.5% Triton X-100 (Sigma-Aldrich) and stained for 30 min using Apollo reaction solution (RiboBio). Upon being washed several times by PBS of 0.5% Triton X-100, cells were stained for 30 min in DAPI (Sigma-Aldrich) in the dark room. Finally, cells were imaged via a microscope (Nikon).

### Flow cytometry for apoptotic cells

Transfected HSC-4 and UM1 cells were firstly collected and washed with PBS. Then, they were stained by using the Annexin V-FITC/PI apoptosis detection kit (BD Biosciences, Franklin Lakes, NJ, USA). Apoptotic rate of the cells was assessed by a flow cytometer and the data collected were analyzed via FACScan (BD Biosciences).

### Transwell migration assay

Migration ability of transfected HSC-4 and UM1 cells was evaluated by utilizing the Transwell System (Costar, Cambridge, MA, USA). Cells in serum-free medium were added to the upper wells, whereas medium with 10% FBS was put to the lower chambers. Twenty-four hours later, cells in the upper layer were removed with caution by a cotton swab and then fixed in methanol solution for 15 min. Crystal violet was adopted to stain the membranes for 10 min, and the invaded or migrated cells were observed and counted under a microscope (10 × 10).

### Western blot analysis

Total protein was extracted from transfected HSC-4 or UM1 cells with RIPA buffer (Beyotime, Shanghai, China). After quantification, total protein was separated by SDS-PAGE (Bio-Rad, Hercules, CA, USA), and electrically transferred onto PVDF membranes (Millipore, Bedford, MA, USA). Membranes were blocked for 1 h employing 5% defatted milk, followed by individually incubated with primary antibodies against Cleaved Caspase-3 (ab2302), Caspase-3 (ab13847), Bax (ab32503), Bcl-2 (ab32124), E-cadherin (ab40772), N-cadherin (ab76057), TPM4 (ab181085) and GAPDH (ab8245) acquired from Abcam (Cambridge, USA). Upon being washed for three times using 0.1 % TBST (Sigma-Aldrich), membranes were incubated for additional 1 h with secondary antibodies. The washing process was repeated and bands were examined by an ECL reagent (Thermo Fisher Scientific).

### Fluorescence in situ hybridization (FISH) assay

HSC-4 and UM1 cells were washed with PBS after being inoculated to glass coverslips in 24-well plates, followed by the fixation of 30 min in 4% formaldehyde (Sigma-Aldrich). After the permeabilization in 70% ethanol (Sigma-Aldrich) overnight, cells were rinsed twice by PBS. Hybridization solution (Sigma-Aldrich) as well as fluorescently labeled NORAD probe (GenePharma) was added during the overnight incubation. Three hours after hybridization, DAPI was adopted to stain cell nuclei and the cells were washed with saline-sodium citrate (SSC; Sigma-Aldrich). In the end, fluorescence microscope was utilized to observe and analyze the stained cells (Olympus Corp., Tokyo, Japan).

### RNA pull-down assay

The Pierce™ RNA3′ End Desthiobiotinylation Kit was utilized for RNA pull-down assay. Cell lysates of HSC-4 and UM1 cells were incubated with Bio-miR-577-Wt/Mut or Bio-NC. Cell protein extracts were then prepared to be mixed with magnetic beads (Invitrogen) and Bio-NC or Bio-miR-577. NORAD or TPM4 level in the complex which was pulled down by biotinylated RNAs was assayed by RT-qpCR analysis.

### Luciferase reporter assay

The wild-type or mutant interacting sequences of miR-577 in NORAD or 3′-UTR of TPM4 were sub-cloned into pmirGLO dual-luciferase vector (Promega, Madison, WI, USA) to establish pmirGLO-NORAD-Wt/Mut or pmirGLO-TPM4-Wt/Mut which was co-transfected into HSC-4 and UM1 cells with miR-577 mimics or NC mimics. To examine luciferase activities, dual luciferase reporter assay system (Promega) was adopted after 48 h.

### RNA immunoprecipitation (RIP) assay

With the RNA-Binding Protein Immunoprecipitation Kit (Millipore, Bedford, MA), RIP assay in HSC-4 and UM1 cells was achieved with the specific antibodies and normal control anti-IgG (Abcam) and anti-Ago2 (Abcam). Lysates were obtained from OSCC cell lines using RIP lysis buffer. The lysis was incubated with the magnetic beads conjugated with the Ago2 antibody or IgG antibody (negative control). The precipitated RNAs were analyzed by RT-qPCR assay.

### Statistical analysis

Data were presented as mean ± SD. All assays were conducted for three times. To analyze the differences between groups, Student’s *t*-test or one-way ANOVA was applied. With the application of Prism 7.0 software (GraphPad, San Diego, CA, USA) or SPSS 17.0 software (SPSS, Chicago, IL, USA), data were analyzed statistically, with the probability (*P*) < 0.05 as threshold.

## Supplementary Information

**Additional file 1: Figure S1.** (A) The expression of TPM4 and miR-577 in OSCC tissues and normal adjacent tissue was detected. (B) The expression of NORAD, miR-577 and TPM4 in OSCC tissues were extracted from online TCGA-HNSC datasets was presented. **P < 0.01.

## Data Availability

Not applicable.

## Abbreviations

lncRNAs: Long non-coding RNAs
OSCC: Oral squamous cell carcinoma
RISCs: RNA-induced silencing complexes
ceRNA: Competing endogenous RNA
miRNAs: MicroRNAs
CCK8: Cell counting kit-8
FISH: Fluorescence in situ hybridization
