# Supplementary figures and images for: LncRNA NORAD facilitates oral squamous cell carcinoma progression by sponging miR-577 to enhance TPM4
Source: Biol Direct. 2022 Jan 6;17:1. doi: 10.1186/s13062-021-00299-2 (PMC8734353; doi:10.1186/s13062-021-00299-2)

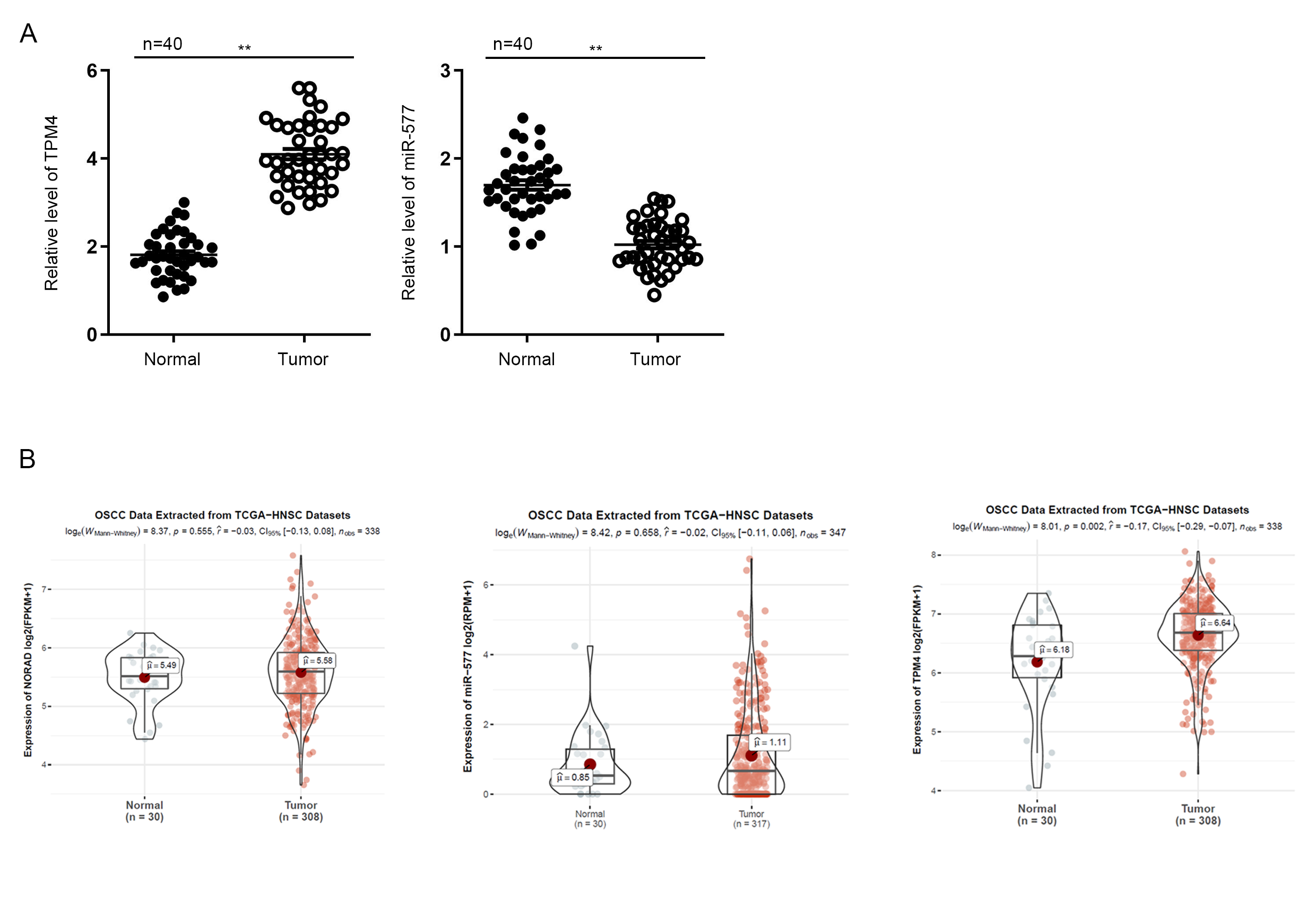

Supplement: Supplementary file 1 — Additional file 1: Figure S1. (A) The expression of TPM4 and miR-577 in OSCC tissues and normal adjacent tissue was detected. (B) The expression of NORAD, miR-577 and TPM4 in OSCC tissues were extracted from online TCGA-HNSC datasets was presented. **P < 0.01. [file 13062_2021_299_MOESM1_ESM.tif]
